# Look me in the eyes: constraining gaze in the eye-region provokes abnormally high subcortical activation in autism

**DOI:** 10.1038/s41598-017-03378-5

**Published:** 2017-06-09

**Authors:** Nouchine Hadjikhani, Jakob Åsberg Johnels, Nicole R. Zürcher, Amandine Lassalle, Quentin Guillon, Loyse Hippolyte, Eva Billstedt, Noreen Ward, Eric Lemonnier, Christopher Gillberg

**Affiliations:** 1 MGH/Martinos Center for Biomedical Imaging, Harvard Medical School, Boston, USA; 2 Gillberg Neuropsychiatry Center, Gothenburg University, 41119 Gothenburg, Sweden; 3 Section for Speech and Language Pathology, Gothenburg University, 41119 Gothenburg, Sweden; 4 Autism Research Centre/Department of Psychiatry, Cambridge University, Cambridge, CB2 8AH UK; 5 Lyon Neuroscience Research Center, Brain Dynamics and Cognition Team, Lyon, France; 6 Service de Génétique Médicale, University of Lausanne, Lausanne, Switzerland; 7 CRA, Limoges, France

## Abstract

Individuals with Autism Spectrum Disorder (ASD) seem to have difficulties looking others in the eyes, but the substrate for this behavior is not well understood. The subcortical pathway, which consists of superior colliculus, pulvinar nucleus of the thalamus, and amygdala, enables rapid and automatic face processing. A specific component of this pathway – i.e., the amygdala – has been shown to be abnormally activated in paradigms where individuals had to specifically attend to the eye-region; however, a direct examination of the effect of manipulating the gaze to the eye-regions on all the components of the subcortical system altogether has never been performed. The subcortical system is particularly important as it shapes the functional specialization of the face-processing cortex during development. Using functional MRI, we investigated the effect of constraining gaze in the eye-region during dynamic emotional face perception in groups of participants with ASD and typical controls. We computed differences in activation in the subcortical face processing system (superior colliculus, pulvinar nucleus of the thalamus and amygdala) for the same stimuli seen freely or with the gaze constrained in the eye-region. Our results show that when constrained to look in the eyes, individuals with ASD show abnormally high activation in the subcortical system, which may be at the basis of their eye avoidance in daily life.

## Introduction

Individuals with autism spectrum disorder (ASD) often report that looking in the eyes of others is uncomfortable for them, that it is terribly stressful, or even that ‘it burns’ (e.g. ref. 1). Although traditional theoretical accounts of ASD have interpreted lack of eye contact and other social difficulties as indicators of interpersonal indifference to others^2^, first hand reports from verbal people with ASD would rather suggest that the underlying problem may be one of socio-affective oversensitivity. Some have even proposed that the amygdala may be hyper-reactive in ASD, resulting in a painfully intense (social) world^3^. As of yet, however, the evidence pertaining to this fundamental issue is limited and mixed. For instance, others have reported what was interpreted as a passive social insensitivity to the eyes of others in a small sample of two year children with autism^4^.

The presence of a subcortical pathway, conveying rapid emotional information via magnocellular inputs from the retina to the superior colliculus, the pulvinar and finally to the amygdala, has been shown in rodents (e.g. ref. 5), in primates (e.g. ref. 6), in blindsight patients (refs 7–11 for review see ref. 12) and in healthy volunteers undergoing subliminal emotional stimulation^13, 14^. Recently, this fast pathway for emotion was directly evidenced for the first time with intracranial electrophysiological data in the human amygdala^15^.

The subcortical system is involved in face processing, in particular in face detection^16^, and it is the starting point for the development of face specialization. It modulates cortical processing and is sensitive to direct gaze (ref. 17 for review, see ref. 18). Newborns’ looking preferences are presumed to be mediated by the subcortical pathway over the first months, and help the normal maturation of the visual cortical areas involved in face perception. This pathway has been thought to be specific for threat, and although fearful face stimuli may serve as optimal stimuli for the subcortical face processing network^19^, it was shown in a blindsight patient that it could also convey other, positive emotions^20^. Moreover, it is known that the subcortical pathway has broad, indirect implications in the adequate execution of social actions through motivation-based attention selection^19^.

Research on the involvement of subcortical brain areas during emotion processing in ASD has yielded mixed findings, with some studies showing absent engagement of subcortical brain regions during emotional face processing (e.g. ref. 21), while others have shown enhanced involvement of these areas^22–24^. One potentially explanatory factor to these mixed findings is eye contact^25^ – that is, whether the subjects attended to eye region of the face stimuli in the experiments or not. Indeed, Dalton *et al.* showed that amygdala activation in ASD children was correlated with spontaneous variations in time spent looking in the eyes of the face^26^. This suggests that some level of experimental control over participants’ gaze patterns may be critical for characterizing the neural substrate of emotional face processing in ASD^27^. No previous study has directly examined the effect of looking in the eyes on subcortical pathway activation in ASD. Nevertheless, Tottenham *et al*.^28^ demonstrated using an elegant paradigm that when ASD participants had to engage in a task that involved detecting a shape placed in the left or the right eye of faces, they showed heightened amygdala activity compared with controls, and that those who in natural settings had the least eye-movements towards the eyes were exhibiting the highest amygdala response when gaze was experimentally driven towards the eyes. In addition, Perlman *et al*.^29^ found, in a study conducted with 12 participants with ASD and 7 controls, that the level of amygdala activity in ASD participants was lower in a free viewing mode compared to controls, but that activity was modulated by experimental manipulation of gaze pattern towards the nose and eyes.

The meaning of direct eye-contact depends on the facial expression of the person, in terms of emotional valence; for instance, a smiling face with a direct gaze is engaging, while an angry face with a direct gaze signals a potential threat^30–34^. Neutral faces are more ambiguous, and they can be perceived as emotionally negatively valenced^35–39^ and even threatening in socially anxious individuals^40, 41^. ASD participants have been shown to have reduced naturally occurring eye-contact to neutral faces, associated with higher threat ratings for these faces^28^. Finally, fearful faces have been shown to automatically attract attention in the eye-region^42^. We decided to examine neutral, happy, angry and fearful faces in our paradigm, and to include all emotions in the analyses to confirm that this is indeed meaningful. In particular, we tested the hypotheses that in each region of the face-processing subcortical pathway, there would be (1) within the ASD group, increased activation in response to constrained viewing (CROSS; i.e. the eye region) compared with the free-viewing (NO-CROSS) condition, and (2) between groups, individuals with ASD would have more activation relative to controls in the constrained (CROSS) viewing condition, and that (3) this effect would be the most marked for fear.

## Results

The aim of the present study was to specifically examine the effect of constraining gaze in the eye-region on activation of the subcortical system in participants with ASD (n = 23) and in matched controls (CON, n = 20), and to test the hypothesis that looking in the eyes would activate rapid emotion-processing pathways in ASD. We used the exact same dynamic facial emotional stimuli in a free-viewing condition and in a condition where participants were specifically asked to look at a cross situated in the eye-region, presented in two separated, counterbalanced runs. (see supplementary information for details).

We defined anatomical ROIs in the superior colliculus, the pulvinar nucleus of the thalamus, and the amygdala, and compared the level of activation for the free-viewing condition (NO-CROSS) and the constrained condition (CROSS) in these ROIs. Left and right amygdala were considered separately as there is evidence for an asymmetric engagement of this structure during face and emotion processing (e.g. refs 43–45). Initially, a 2 (Group: ASD, CON) by 4 (Emotion: Neutral, Happy, Angry, Fear) by 2 (Condition: NO-CROSS, CROSS) by 4 (ROI: left amygdala, right amygdala, pulvinar, superior colliculus) mixed factorial analysis of variance (ANOVA) was performed. The full four-way interaction proved significant (*F*_1,6.41_ = 2.22, p = 0.038, *η*_*p*_^2^ = 0.051), which motivates the next step in the analyses where separate ANOVAs for each ROI with planned comparisons between and within groups for each emotion and for each condition were carried out. In addition, we tested that regardless of emotion, within the ASD group, there would be increased activation in response to constrained viewing (CROSS; i.e. in the eye region) compared with the free-viewing (NO-CROSS) condition, and that between group, individuals with ASD would have more activation relative to controls in the constrained (CROSS) viewing condition.

**Figure 1:**
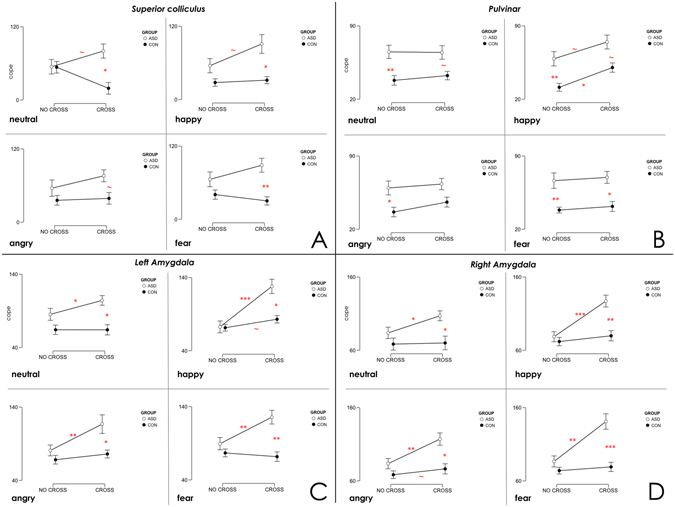
Descriptive plots of the ANOVA for each of the four ROIs. Panel A: Superior colliculus; Panel B: Pulvinar; Panel C: Left amygdala; Panel D: Right amygdala. Each panel shows the results obtained for neutral, happy, angry and fear faces. ASD participants are shown in white, controls (CON) in black. Values represented indicate mean ± SEM. Significant differences are indicated by red symbols (~trend, *p < 0.05; **p < 0.01; ***p < 0.001).

In the superior colliculus and both amygdalae, ASD and controls had the same amount of activation in the free-viewing condition, but the ASD group had abnormally high activation compared to controls when constrained to look in the eyes of neutral and emotional faces. In the pulvinar, ASD participants had higher activation than controls for both freeviewing and constrained viewing, with exception of angry where, when forced to look into the eyes, there was no significant difference between groups (Fig. 1) (see supplementary information for details). Within the ASD group, the effect of adding a fixation cross in the eye-region was most evident in both amygdalae where activation patterns that were consistently higher compared with the NO-CROSS condition.

We then tested the hypothesis that the size of the effect for the between group comparison in the CROSS condition would be the most important for FEAR. We computed Cohen’s d for each ROI and for each condition, and found that the maximum effect size was always for the FEAR condition (see supplementary information for details).

Finally, we tested that autism symptom severity, as measured by AQ, would be positively correlated with activation in the subcortical system in ASD, and found such a correlation for FEAR in all subcortical areas, and for NEUTRAL in three of the four areas, in the free-viewing condition. See supplementary information for details.

## Discussion

Our data demonstrate that constraining individuals with ASD to look into the eyes of dynamic faces expressing different emotions results in aberrant activation of the subcortical pathway, such that higher activation was found generally in the ASD group. An abnormality of the subcortical system in autism during face processing was first hypothesized by Senju and Johnson^25^, and our data not only confirm this hypothesis, but specify it to a considerable degree: our direct comparison of the same dynamic facial expression seen freely or with a fixation cross is the most direct evidence of the mechanisms by which direct eye-contact may be experienced as stressful in autism.

ASD participants had higher pulvinar activation than controls in both conditions, and it seems that activity in this structure is less consistently modulated by eye contact. The pulvinar can be considered as a central forebrain hub and its input from the superior colliculus may be critical in shaping the functional specialization of the cortex during early development (for review, see ref. 46). One of the roles of the pulvinar is to filter distracting stimuli^47^, and recently morphological alterations of this structure have been reported in ASD, with an expanded surface area^48^. The thalamic hyperactivation evidenced here has been hypothesized as one of the substrates of higher-order social cognition deficits in ASD, potentially through its dysregulatory impact on the dorsolateral prefrontal cortex^49^. The role of the pulvinar in both the higher-order cognitive and the basic socio-affective profile in ASD will need to be examined in future studies.

As could be expected from previous studies (e.g. ref. 26), the effect of constraining gaze in the eyes had the strongest effect for ASD in the amygdala, and although group differences were most marked for fearful faces, the difference between free and constrained gaze was also remarkable for happy faces. This shows that the subcortical system in ASD over-reacts not only to threat-related stimuli, but also to stimuli that should be considered as positively engaging and socially rewarding.

Our findings deepen our understanding of the mechanisms at play in the social deficits of individuals with autism. Traditional accounts have suggested that ASD is characterized by a fundamental lack of interpersonal interest^2^; however, the results of our study align with other recent studies showing oversensitivity to socio-affective stimuli (e.g. refs 50, 51). In everyday life, such oversensitivity may lead to attempts to decrease one’s arousal levels, and first-hand reports suggest that simply avoiding to attend to the eyes of others is one common strategy among individuals with ASD^1^. Such a strategy is unlikely, however, to come without costs, since the eyes carry important interpersonal and deictic information during social interaction and communication, and eye-avoidance may result in cascading effects leading to improper development of the social brain.

### Limitations

There are several limitations in this work. First, we did not collect eye-tracking data in the scanner, and hence we never directly correlated subcortical brain activation with the amount of time spent in the eye-region^26^. However, while interesting, the relative merits of that approach should not be overstated. Indeed, abnormal gaze patterns in ASD during spontaneous face viewing has been demonstrated repeatedly, and although not all studies find evidence of reduced eye gaze in ASD samples, more robust differences in how they distribute their attention within the central areas of the face has been described^52, 53^. Moreover, while there are current developments in fixation-based^54^ and event-related^55^ fMRI, challenges still exists when trying to associate a hemodynamic *response* (that lasts ca 10 *seconds*) with fixation-based metrics (which averages 300 msec during scene viewing)^54^.

Second, we only tested constrained gaze with a face, and not with a blank screen or with non-face stimuli, so part of our results may be due to a general effect of constraining gaze. One can perhaps expect that fixating a cross might introduce some level of cognitive control in the task. Future research should use free-viewing vs. constrained gaze for non-face stimuli as well, so as to better determine whether part of the effect observed can be attributed to this factor. Still, there are to our knowledge no theoretical or empirical reasons to predict an enhanced activation of the face-specific subcortical system following a fixed gaze in general.

Third, although we and others have plenty of clinical evidence for the notion that many individuals with ASD find eye contact stressful, no research study, including the one presented, have actually linked subjective reports of discomfort with neural activity patterns during eye gaze. This would be an important area of future research (though one also needs to hold in mind the potential limitations of self-reports in a condition that is very often associated with alexithymia)^56^.

Finally, as is most often done in this kind of technically challenging studies, we only examined participants with normal intelligence, so we do not know whether these results generalize to the full spectrum of ability in the ASD population. We hope that further studies using less invasive techniques such as eye-tracking with galvanic skin response will help us investigate a wider range of ASD individuals.

Despite these caveats, the results have potential clinical implications: during behavioral therapy, forcing individuals with autism to look in the eyes might be counterproductive and elicit more anxiety - however, by not looking at the eyes, the person with ASD will continue to miss critical social information, and somehow one has to help them to gather all these important cues. One possible strategy could consist in progressively habituating individuals with ASD to look into the eyes, analogous to the way surgeons habituate to look at open bleeding bodies, and then in incentivizing them to look at the eyes, finding a way to make eye contact somehow less stressful.

## Methods

### Participants

All procedures were in accordance with the Declaration of Helsinki and were approved locally by the Lausanne University Hospital Ethics Committee. Written informed consent was obtained from all adult participants and from all parents of participating children, before the start of the study. In addition, all children participants gave their oral assent to partake in the study.

Twenty-five ASD participants were enrolled in the study. Only participants (ASD and CON) who had an estimated absolute mean displacement of less than 2 mm as reported by FLS MCFLIRT and who responded 75% or more to the monitoring procedure that we used to track if the participants paid attention were included. Two ASD subjects were excluded from the data analysis due to excessive movement (n = 1) or for not performing the task during the scan (n = 1). Thus, 23 ASD participants (21 males, 22.6 years ± 1.8 (mean age ± SEM), range 10.6–40.7) were included in the final analysis.

Participants with ASD were all diagnosed by experienced clinicians certified reliable for research purposes who used the DSM IV-TR criteria^57^, together with the Autism Diagnostic Interview-Revised (n = 17)^58^ or the Diagnostic Interview for Social and Communication Disorders (DISCO) version 10 or 11^59^ (n = 5), and the Autism Diagnostic Observation Schedule module 4 (n = 15) or module 3 (n = 3)^60^. Of the 23 participants, 16 had a diagnosis of autism, 5 of Asperger Syndrome and 2 of PPD-NOS.

Twenty-five healthy control participants (CON) were enrolled in the study. They had no history of psychiatric or neurological disorders. Five subjects were excluded from the data analysis due to excessive movement (n = 3) or for not performing the task during the scan (n = 2). Thus, 20 control participants (17 males, 23.3 years ± 1.8 (mean age ± SEM), range 12.7–42.9) were included in the final analysis.

Intelligence Quotient (IQ) scores were obtained using the Wechsler Nonverbal Scale of Ability (WNS)^61^ or the Wechsler Abbreviated Scale of Intelligence (WASI)^62^. All participants had normal IQ (ASD: 112.9 ± 3.3; CON: 113.4 ± 2.4 (mean ± SEM)). ASD and CON participants were matched for age and IQ. (*p* > 0.7).

Participants also completed the Autism-Spectrum Questionnaire^63, 64^. (ASD: 26.8 ± 1.4; CON: 13.3 ± 1.5 (mean ± SEM)). ASD had significantly higher scores than CON (*p* < 0.001).

Anxiety levels were evaluated with the State-Trait Anxiety Inventory (STAI)^65^ in 12 ASD and 15 CON adults (data were not collected for 5 ASD adults), and the Revised Children’s Manifest Anxiety Scale RCMAS in 6 ASD and 5 CON children^66^. The groups did not differ on these scores (ASD STAI trait: 53.33 ± 12.46; CON STAI trait: 46.13 ± 6.78, t-test: p = 0.09; RCMAS score ASD: 55.00 ± 11.31; CON: 54.60 ± 6.87 T test: p = 0.94).

### Experimental design

Twenty-four movies were created from the NimStim database^67^ representing morphs of facial expressions from NEUTRAL to FEAR, HAPPY or ANGER with Morph Age Pro (Creaceed). Each movie lasted for 5 seconds, and consisted of a dynamic morph lasting 3 seconds, followed by 2 seconds of the final emotional expression. Morphs of NEUTRAL were also created by creating a left-to-right morph between mirror images of neutral faces, in order to also have a dynamic component in this condition. Two versions of these movies were created, with one version containing a red fixation cross in the region of the eyes. The NimStim database does not allow to publish the identities that we used in the experiment, but we created a representative example from one of the authorized identities that can be consulted in the supplementary material. In addition, a red fixation cross was present for 1 second between each movie at the same location, and periods of FIXATION were presented for 6 seconds at the beginning and at the end of each run, as well as for 3 seconds 7 times during each run interspersed between the blocks of emotional faces. Each participant viewed both versions (CROSS and NO-CROSS) during the scanning session (that also comported other tasks not reported in the present manuscript). The order of the CROSS and NO-CROSS versions was counterbalanced across participants, so that about half of them saw the stimuli with NO-CROSS first. Participants were instructed to carefully look at the videos and, in order to monitor their attention, to press a button every time they saw a blue cross between the stimuli, which happened 4 times during CROSS and 4 times during NO-CROSS. The stimuli presented during CROSS and NO-CROSS were identical, the only difference was the presence of a fixation cross during the CROSS version. Each block lasted 48 seconds. There were 8 blocks (2 for each emotion) and within one block, here were 8 stimuli (all different identities) for a total of 16 stimuli per condition.

### Imaging data acquisition and analysis

Anatomical and functional MR images were collected in all participants with a 12-channel RF coil in a Siemens 3 T scanner (Siemens Tim Trio, Erlangen). T1-weighted anatomical images were acquired using an ME-MPRAGE (176 slices; 256 × 256 matrix; 1 × 1 × 1 mm voxels, echo time (TE): TE1: 1.64 ms, TE2: 3.5 ms, TE3: 5.36 ms, TE4: 7.22 ms; repetition time (TR): 2530 ms; flip angle 7°). Functional data were obtained using an echo planar imaging (EPI) sequence (47 AC-PC slices, 3 × 3 × 3 mm voxels, 64 × 64 matrix; FOV: 216; TE: 30 ms; TR: 3000 ms; flip angle 90°) lasting 384 seconds.

Functional MRI data processing, as well as preprocessing was carried out using FSL 5.0.2.2. Non-brain tissue was removed from high-resolution anatomical images using Christian Gaser’s VBM8 toolbox for SPM8^68^ and fed into feat. Data were motion-corrected using MCFLIRT and motion parameters added as confound variables to the model. Participants with motion exceeding 2mm were excluded from further processing (1 ASD, 3 controls). Paired t-test within each group comparing average head movements in the CROSS and NO-CROSS conditions were not significant (ASD: t_22_ = 1.55, p = 0.135; CON: t_19_ = 0.77, p = 0.447). Unpaired t-tests between group for each condition were not significant either (NO-CROSS: t_41_ = 0.83, p = 0.41; CROSS: t_41_ = 1.26, p = 0.21). Residual outlier timepoints were identified using FSL’s motion outlier detection program and integrated as additional confound variables in the first-level General Linear Model (GLM) analysis. Preprocessing included spatial smoothing using a Gaussian kernel of 8 mm, grand-mean intensity normalization and highpass temporal filtering with sigma = 50.0 s.

Subject-level statistical analysis was carried out for the contrasts [NEUTRAL > FIXATION], [HAPPY > FIXATION], [ANGRY > FIXATION and [FEAR > FIXATION] using FILM with local autocorrelation correction for both the CROSS and the NO-CROSS runs. Registration to high-resolution structural images was carried out using FLIRT. Registration to MNI standard space was then further refined using FNIRT nonlinear registration. Group-level analyses for each condition were performed using mixed effects GLM analysis using FLAME 1 + 2 with automatic outlier detection.

The regions of interest (ROIs) in the subcortical system were anatomically defined and consisted of the left and right amygdala (from the Harvard-Oxford Subcortical atlas), the superior colliculus and the pulvinar nucleus of the thalamus. For each subject, the value of the maximum contrast of parameter estimate (COPE) was extracted for the four structures and the four contrasts of interest, using the FSL Featquery tool in FSL.

## Electronic supplementary material

Supplementary Information

Angry No Cross

Angry Cross

Fear No Cross

Fear Cross

Happy No Cross

Happy Cross

Neutral No Cross

Neutral Cross
