# Supplementary material for: Look me in the eyes: constraining gaze in the eye-region provokes abnormally high subcortical activation in autism
Source: Sci Rep. 2017 Jun 9;7:3163. doi: 10.1038/s41598-017-03378-5 (PMC5466661; doi:10.1038/s41598-017-03378-5)
Supplement: Supplementary file 1 — Supplementary Information [file 41598_2017_3378_MOESM1_ESM.pdf]

Look me in the eyes: constraining gaze in the eye-region provokes abnormally high subcortical activation in autism.

Nouchine Hadjikhani (MD, PhD)1,2,*, Jakob Åsberg Johnels (PhD)2,3, Nicole R Zürcher (PhD)1, Amandine Lassalle (PhD)1,4, Quentin Guillon (PhD)5, Loyse Hippolyte (PhD)6, Eva Billstedt (PhD)2, Noreen Ward (MSc)1, Eric Lemonnier (MD, PhD)7, Christopher Gillberg (MD, PhD)2.

Supplementary Information:

Results:

Initially, an omnibus 2 (Group: ASD, CON) by 4 (Emotion: Neutral, Happy, Angry, Fear) by 2 (Condition: No Cross, Cross) by 4 (ROI: left amygdala, right amygdala, pulvinar, superior colliculus) mixed factorial analysis of variance (ANOVA) was performed. The Greenhouse-Geisser correction was used for correcting against violations against sphericity The full four-way interaction effect was significant ($F_{1,6.41}$ = 2.22, p = 0.038, η_p^2 = 0.051) . Following this result, 2 (Group: ASD, CON) by 4 (Emotion: Neutral, Happy, Angry, Fear) by 2 (condition: No Cross, Cross) mixed factorial ANOVAs were performed separately for each ROI, and followed up by two-tailed planned comparisons between groups for each Emotion for the CROSS and the NO CROSS conditions separately, as well as within the groups comparing the CROSS and the NO CROSS conditions for each Emotion.

Superior colliculus: There was a main effect of Group, where ASD showed more activation than CON ($F_{1,41}$=6.606, p=0.014, η_p^2 = 0.139). In addition, there was a trend for a Condition * Emotion interaction ($F_{3,123}$=2.627, p=0.053, η_p^2 = 0.060). Across all Emotions, the effect of Group was not significant for the NO CROSS condition ($F_{1,41}$=1.244). It was however very

significant for the CROSS condition ($F_{1,41}$=7.543, p=0.009,η_p^2 = 0.155). Planned comparisons show significant higher activation for ASD compared with CON for Neutral (p=0.0048), Happy (p=0.0061), and Fear (p=0.0069). There was a trend for Angry (p=0.085). The effect of Condition was not significant for ASD ($F_{1,22}$=2.367, p=0.138, η_p^2 = 0.097), nor for CON ($F_{1,19}$=0.662, p=0.426, η_p^2 = 0.034). Planned comparison showed a trend for higher activation for the CROSS condition in ASD for Neutral (p=0.092) and for Happy (p=0.066) but not for Angry (p=0.142) nor for Fear (p=0.137). None of the planned comparisons were significant in the CON group.

Pulvinar: There was a main Group effect, where ASD showed more activation than CON ($F_{1,41}$=8.107, p=0.007, η_p^2 = 0.165). In addition, there was a significant Condition * Emotion interaction ($F_{3,123}$=3.182, p =0.026, η_p^2 = 0.072). Across all Emotions, the effect of Group was very significant for the NO CROSS condition ($F_{1,41}$=9.322, p =0.004,η_p^2 = 0.185), and post-hoc comparisons show significant higher activation for ASD compared with CON for Neutral (p=0.0081), Happy (p=0.0080), and Angry (p=0.0250) and Fear (p=0.0063). One-way ANOVA was marginally significant for the CROSS condition ($F_{1,41}$=3.854, p=0.056, ,η_p^2 = 0.086). Planned comparisons show significant higher activation for ASD compared with CON only for Fear (p=0.0290*).* The effect of Condition for each Group was not significant for ASD ($F_{1,22}$=0.792, p=0.383, η_p^2 = 0.035). It was significant for CON ($F_{1,19}$=5.398, p=0.031, η_p^2 = 0.221). Planned comparison showed a trend for higher activation for the CROSS condition in ASD (p=0.089) and was significant in CON (p=0.005) for Happy but not for the other emotions.

Left Amygdala: There was a main Group effect, where ASD showed more activation than CON ($F_{1,41}$=5.549, p=0.023, η_p^2 = 0.119). There was also a main effect of Condition

($F_{1,41}$=7.219, p=0.010, η_p^2 = 0.150), of Emotion ($F_{3,123}$=2.942, p=0.036, η_p^2 = 0.067), a Condition * Group interaction ($F_{1,41}$=4.878, p=0.033, η_p^2 = 0.106) as well as a Condition * Emotion interaction ($F_{3,123}$=3.085, p=0.030, η_p^2 = 0.070). Across all Emotions, the effect of Group was not significant for the NO CROSS condition ($F_{1,41}$=0.876). It was however very significant for the CROSS condition ($F_{1,41}$=8.357, p=0.006, η_p^2 = 0.169). Planned comparisons show significant higher activation for ASD compared with CON for Neutral (p=0.0240), Happy (p=0.0118), Angry (p=0.0219) and Fear (p=0.0026). The effect of Condition for each Group was significant for ASD ($F_{1,22}$=9.129, p=0.006, η_p^2 = 0.293), but not for CON ($F_{1,19}$=2.293, p=0.146, η_p^2 = 0.108). Planned comparison showed higher activation for the CROSS condition in ASD for Neutral (p=0.047), Happy (p<0.001), Angry (p=0.023) and Fear (p=0.013); there was a trend in CON (p=0.058) for Happy but not for the other emotions

Right Amygdala: There was a main effect of Group, where ASD showed more activation than CON ($F_{1,41}$=4.738, p=0.035, η_p^2 = 0.104). There was also a main effect of Condition ($F_{1,41}$=9.903, p=0.003, η_p^2 = 0.195), of Emotion ($F_{3,123}$=3.94, p=0.010; η_p^2 = 0.088), as well as a Condition * Group interaction ($F_{1,41}$=5.617, p=0.023; η_p^2 = 0.120). Across all Emotions, the effect of Group was not significant for the NO CROSS condition($F_{1,41}$=0.668). It was however very significant for the CROSS condition ($F_{1,41}$=8.977, p<0.005,η_p^2 = 0.180). Planned comparisons show significant higher activation for ASD compared with CON for Neutral (p=0.0359), Happy (p=0.0076), Angry (p=0.0217) and Fear (p=0.0005). The effect of Condition for each Group was significant for ASD ($F_{1,22}$=13.076, p=0.002, η_p^2 = 0.373), but not for CON ($F_{1,19}$=0.398, p=0.536, η_p^2 = 0.021). Planned comparison showed higher

activation for the CROSS condition in ASD for Neutral (p=0.027), Happy (p<0.001), Angry (p=0.007) and Fear (p<0.001); no planned comparison was significant for CON.

Supplementary Table 1 reports the effect of ANOVA for the different conditions in each structure.

In order to test whether our effect was specific to the face-processing subcortical system, we also analyzed activation in all the subcortical components of the Harvard-Oxford atlas outside the fast-processing subcortical pathway, namely, left/right hippocampus, caudate, putamen, pallidum, and accumbens, respectively. First, we conducted a similar ANOVA as done with the ROIs where we had an *a priori* hypothesis, namely a a 2 (Group: ASD, CON) by 4 (Emotion: Neutral, Happy, Angry, Fear) by 2 (Condition: No Cross, Cross) by 10 (ROI: left hippocampus, right hippocampus, left caudate, right caudate, left putamen, right putamen, left accumbens, right accumbens). The full four-way interaction was not significant ($F_{27, 15}$=0.5, p = 0.94). Yet, we performed further separate ANOVAS between and within groups for each emotion and each condition.

We observed a GROUP effect in the left accumbens ($F_{1,41}$=5.95, p=0.019, η_p^2 = 0.127) and in the right pallidum ($F_{1,41}$=6.001, p=0.019,η_p^2 = 0.128) but in none of the other 8 ROIs. There was no effect of CONDITION in any of the ROIs with the exception of the left accumbens ($F_{1,41}$=9.003, p=0.005,η_p^2 = 0.180) and the right putamen ($F_{1,41}$=6.77, p=0.013,η_p^2 = 0.142). We did not correct for multiple comparisons in the results above. If this is done (80 comparisons in total), none of the results above are close to significant. Neither was there an effect of EMOTION in any of the areas, of CONDITION * GROUP, or of EMOTION * GROUP

(uncorrected for multiple comparisons), indicating that our findings were specific to the subcortical face-processing pathway.

To test that the effect of the CROSS condition between groups was the most marked for the FEAR condition, we computed Cohen's d for each ROI. Our results, illustrated in Figure S1 indicate that FEAR was the emotion in which constraining gaze in the eye-region had the strongest effect in subcortical face-processing activation in ASD compared with controls.

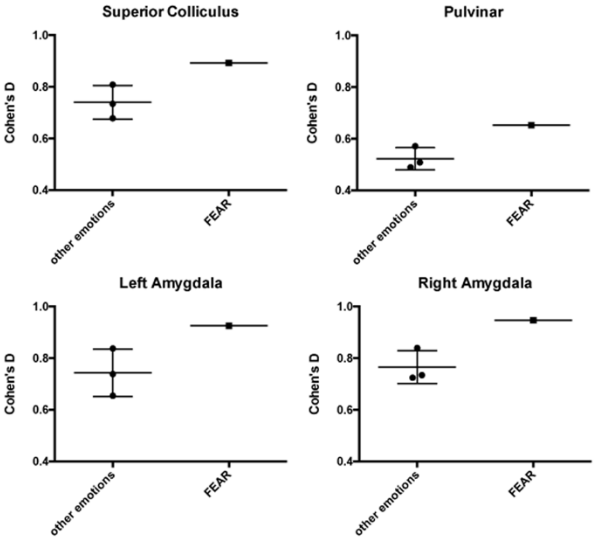

Supplemental Figure 1 shows Cohen's D values for the comparison between ASD and CON in the CROSS condition, for FEAR vs. all other emotions.

We finally tested the hypothesis that autism symptom severity, as measured by AQ, would be positively correlated with activation in the subcortical system in ASD (n=23, df=21). We found positive correlations between AQ and subcortical brain activation in the free-viewing (NO CROSS) condition for FEAR in all subcortical areas and for NEUTRAL in three of the

four areas. This positive correlation in the free-viewing condition only may be counterintuitive at first glance, seemingly contradicting the findings from the diagnostic group comparison. However, we think that it reflects a ceiling level of activation in the constrained gaze condition in the diagnosed ASD group. There were no statistically significant correlations in any of the areas in the CROSS condition in the ASD group. Details of the correlations are given in **Supplementary Table 2.**

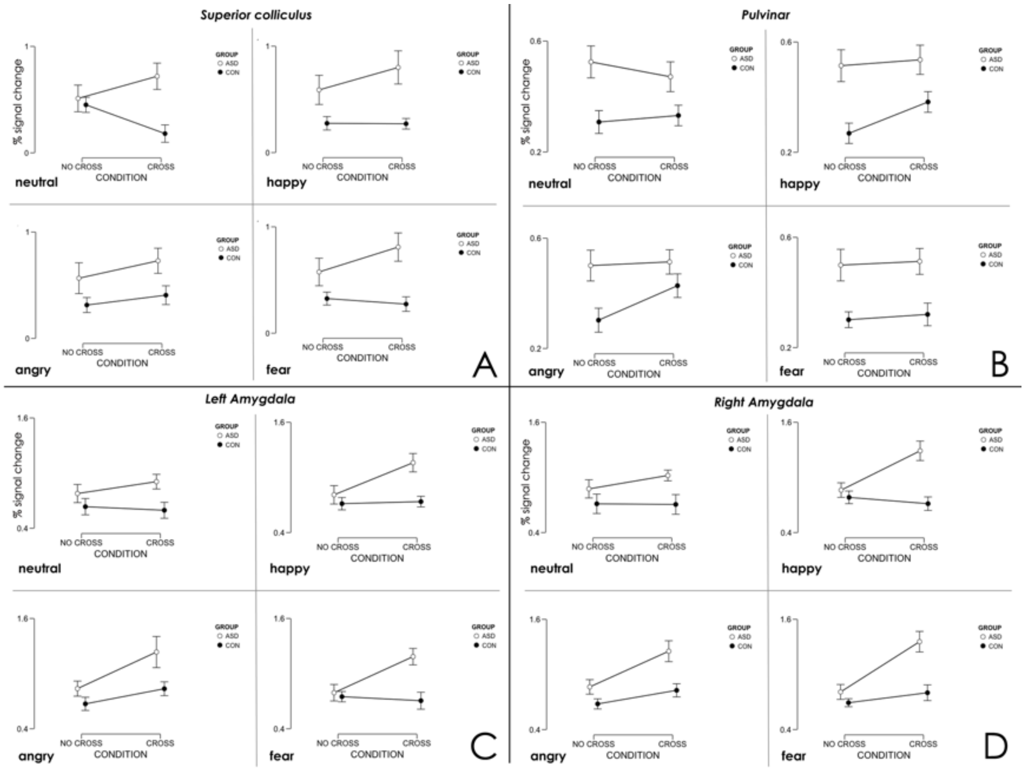

Supplemental Figure 2 shows the results expressed in % signal change instead of COPE.

Supplementary Videos – stimuli

Happy.mov: stimulus used for the happy free-viewing condition (NO CROSS)

This video is not covered by the CC BY licence. [Credits to Tottenham et al., 2009]. All rights reserved, used with permission.

Happy_cross.mov: stimulus used for the happy constrained condition (CROSS)

This video is not covered by the CC BY licence. [Credits to Tottenham et al., 2009]. All rights reserved, used with permission.

Angry.mov: stimulus used for the angry free-viewing condition (NO CROSS)

This video is not covered by the CC BY licence. [Credits to Tottenham et al., 2009]. All rights reserved, used with permission.

Angry_cross.mov: stimulus used for the angry constrained condition (CROSS)

This video is not covered by the CC BY licence. [Credits to Tottenham et al., 2009]. All rights reserved, used with permission.

Fear.mov: stimulus used for the fear free-viewing condition (NO CROSS)

This video is not covered by the CC BY licence. [Credits to Tottenham et al., 2009]. All rights reserved, used with permission.

Fear_cross.mov: stimulus used for the fear constrained condition (CROSS)

This video is not covered by the CC BY licence. [Credits to Tottenham et al., 2009]. All rights reserved, used with permission.

Neutral.mov: stimulus used for the neutral free-viewing condition (NO CROSS)

This video is not covered by the CC BY licence. [Credits to Tottenham et al., 2009]. All rights reserved, used with permission.

Neutral_cross.mov: stimulus used for the neutral constrained condition (CROSS)

This video is not covered by the CC BY licence. [Credits to Tottenham et al., 2009]. All rights reserved, used with permission.

Note: As mentioned, one previous study (Kleinhans *et al.*, 2011) examined subcortical activation in ASD during fearful face perception, and found results opposite to those presented here, namely a hypoactivation of the subcortical system. We interpret this discrepancy, in addition to technical differences (spatial resolution and lower magnetic field in (Kleinhans *et al.*, 2011)) as differences in study design and experimental paradigm: while we use dynamic movies with different facial expression, Kleinhans et al. (Kleinhans *et al.*, 2011) had static fearful faces very briefly presented (23 ms), and the fixation cross was only presented between stimuli, and was not located at the level of the eye-region; ASD participants may therefore not have been attending to the eyes in the stimuli. The fact that in that study only 54% of ASD participants reported seeing fearful faces as well as the lower FFA activation observed in ASD supports this interpretation.

References for Supplementary Material:

Kleinhans, N. M., Richards, T., Johnson, L. C., Weaver, K. E., Greenson, J., Dawson, G. & Aylward, E. (2011) 'fMRI evidence of neural abnormalities in the subcortical face processing system in ASD', *Neuroimage*, **54**(1), pp. 697-704.

Tottenham, N., Tanaka, J. W., Leon, A. C., McCarry, T., Nurse, M., Hare, T. A., Marcus, D. J., Westerlund, A., Casey, B. J. & Nelson, C. (2009) 'The NimStim set of facial expressions: judgments from untrained research participants', *Psychiatry Res*, **168**(3), pp. 242-249.

	superior colliculus	pulvinar	left amygdala	right amygdala
GROUP	**$F_{1,41}$=6.606, p=0.014, η_p^2 = 0.139**	**$F_{1,41}$=8.107, p=0.007, η_p^2 = 0.165**	**$F_{1,41}$=5.549, p=0.023, η_p^2 = 0.119**	**$F_{1,41}$=4.738, p=0.035, η_p^2 = 0.104**
CONDITION	$F_{1,41}$=0.536, p=0.47,η_p^2 = 0.013	$F_{1,41}$=1.774, p=0.19,η_p^2 = 0.041	**$F_{1,41}$=7.219, p=0.01,η_p^2 = 0.136**	**$F_{1,41}$=9.903, p=0.003,η_p^2 = 0.175**
CONDITION*GROUP	$F_{1,41}$=2.430, p=0.127,η_p^2 = 0.056	$F_{1,41}$=0.102, p=0.75,η_p^2 = 0.002	**$F_{1,41}$=4.878, p=0.033,η_p^2 = 0.072**	**$F_{1,41}$=5.617, p=0.023,η_p^2 = 0.099**
EMOTION	$F_{3,123}$=0.387, p=0.76, η_p^2 = 0.009	$F_{3,123}$=0.337, p=0.80, η_p^2 = 0.008	**$F_{3,123}$=2.945, p=0.036, η_p^2 = 0.066**	**$F_{3,123}$=3.940, p=0.010, η_p^2 = 0.064**
EMOTION*GROUP	$F_{3,123}$=1.052, p=0.37, η_p^2 = 0.025	$F_{3,123}$=0.593, p=0.62, η_p^2 = 0.014	$F_{3,123}$=0.592, p=0.62, η_p^2 = 0.013	$F_{3,123}$=0.899, p=0.44, η_p^2 = 0.020
CONDITION*EMOTION	**$F_{3,123}$=2.627, p=0.053, η_p^2 = 0.060**	**$F_{3,123}$=3.182, p =0.026, η_p^2 = 0.072**	**$F_{3,123}$=3.085, p=0.030, η_p^2 = 0.070**	$F_{3,123}$=1.907, p=0.132, η_p^2 = 0.043
GROUP effect for CROSS	**$F_{1,41}$=7.543, p=0.009,η_p^2 = 0.155**	**$F_{1,41}$=3.854, p=0.056, ,η_p^2 = 0.086**	**$F_{1,41}$=8.357, p=0.006, η_p^2 = 0.169**	**$F_{1,41}$=8.977, p<0.005,η_p^2 = 0.180**

Supplementary Table 1: Results of the one-way ANOVA for each structure

	NEUTRAL NO CROSS	HAPPY NO CROSS	ANGRY NO CROSS	FEAR NO CROSS	NEUTRAL CROSS	HAPPY CROSS	ANGRY CROSS	FEAR CROSS
**Superior colliculus**								
Spearman's rho	0.135	0.078	0.104	**0.38**	0.24	*0.325*	0.178	0.161
p-value	0.27	0.361	0.318	**0.037**	0.135	*0.065*	0.208	0.232
**Pulvinar**								
Spearman's rho	**0.53**	0.249	0.235	**0.424**	0.227	0.216	0.192	0.195
p-value	**0.005**	0.126	0.14	**0.022**	0.149	0.161	0.19	0.186
**Left Amygdala**								
Spearman's rho	**0.471**	*0.342*	*0.313*	**0.409**	*0.309*	0.15	0.159	0.166
p-value	**0.012**	*0.055*	*0.073*	**0.026**	*0.076*	0.248	0.234	0.224
**Right Amygdala**								
Spearman's rho	**0.352**	0.056	0.06	0.393	*0.307*	0.142	0.168	0.059
p-value	**0.05**	0.401	0.393	**0.032**	*0.077*	0.259	0.222	0.395

Supplementary Table 2: Positive correlations between AQ and activation in the subcortical system in the ASD group (n=23, df=21). Significant correlations are in bold, and trends are in italics.
